# Supplementary material for: Direct observation of a superconducting vortex diode
Source: Nat Commun. 2023 Mar 24;14:1630. doi: 10.1038/s41467-023-37294-2 (PMC10036628; doi:10.1038/s41467-023-37294-2)
Supplement: Supplementary file 1 — Supplementary information [file 41467_2023_37294_MOESM1_ESM.pdf]

Supplementary Information for "Direct Observation of a Superconducting Vortex Diode"

Supplementary Figures

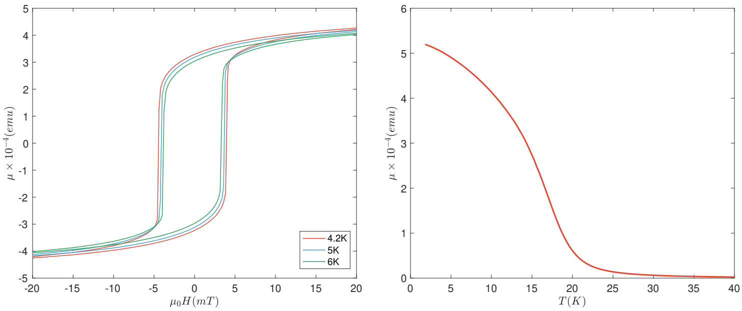

Supplementary Fig. S1 Global magnetization measurements. **a** In-plane $M(H)$ curves of an unpatterned EuS/Nb film with the same thicknesses as our device for different temperatures. The red curve was taken at 4.2K, the blue curve at 5K and the green curve at 6K.**b** $M(T)$ curve of the same device. An in-plane field of 50mT was applied for this measurement

Supplementary Note I

Defining the critical current by the I-V slope

The following note provides a detailed explanation about the method used for defining the critical currents from which the the asymmetry factor presented in Fig. 3a of the main paper was calculated. The criterion used was to impose a very small threshold on the derivative of the $I-V$ curves in order to 'detect' already a very slight increase in voltage. We note, that this criterion may not provide a well defined value of the physical critical current which gives rise to macroscopic flux flow, but rather gives a parameter that qualitatively represents the asymmetry of the transport curves. Furthermore, in order to produce

clear results that focus mainly on the 3 different states ($H > H_{s+}$,$H < -H_{s-}$ and $H \approx H_c$) we smooth the raw data by applying a linear regression filter and define the threshold to be $20m\Omega$. Supplementary Fig. S2 shows explicitly the derivative of a single $I - V$ curve after applying the filter. I_{c+} and I_{c-} are obtained by the intersection of the plotted curve with the threshold of $20m\Omega$.

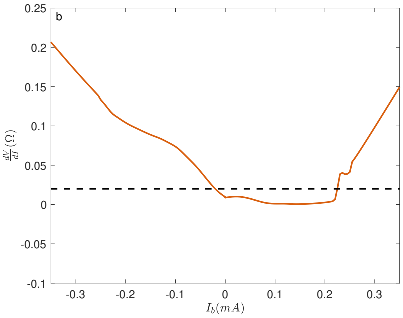

Supplementary Fig. S2 Analysis of the asymmetry factor by the derivative criterion. $\frac{dV}{dI}$ of one $I - V$ curve. A linear regression filter was applied to the raw data in order to produce a smooth derivative. The dashed line represents the threshold of $20m\Omega$

Defining the critical current by voltage threshold

It is also possible to perform this analysis by assuming a threshold on the voltage value in the $I - V$ curves. In this case, no smoothing of the data is necessary and we can assume a threshold of $2\mu V$. The result of this method of analysis is shown in Supplementary Fig. S3a. The resulting data points show exactly the same trend as those achieved by the derivative criterion (Fig. 3a of the main article), but they are far more scattered and can deflect the attention from the main observation.

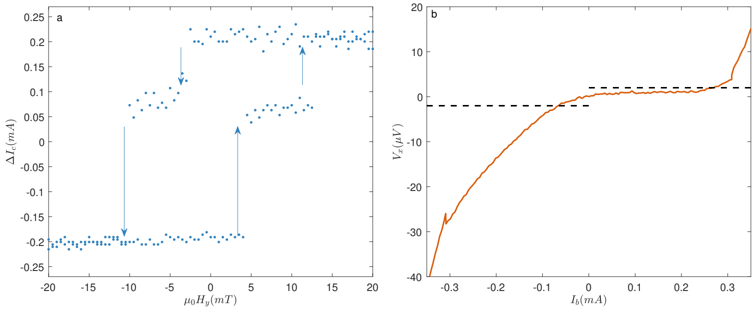

Supplementary Fig. S3 Analysis of the asymmetry factor by the voltage criterion. (**a**) Asymmetry factor $\Delta I_c = |I_c^+| - |I_c^-|$ as a function of transverse magnetic field $\mu_0 H_y$. The arrows mark the magnetic field sweep directions. (**b**) Example of one $I - V$ curve with the chosen threshold of the voltage (dashed black line).

Supplementary Note II

Calculation of the Screening Currents

Here we provide details of the calculation of the screening current in the S layer induced by a stray magnetic field of the F layer where we neglect the superconducting proximity effect and electromagnetic interaction between the layers. For the limit $L \gg d_f$ the stray field in the region of the superconductor layer can be expressed with the help of a vector potential $A_{\rm M}(y)$. The field $\vec{\mathbf{H}}$ can be modeled by the field induced by two infinite wires with opposite magnetic charge densities $\pm M d_f$ positioned at $y = \pm L/2$ and $z = 0$. Note here that the plane $z = 0$ is chosen to pass through the middle of the F layer. We also neglect the finite thickness of the S strip and consider it as a delta layer placed in the $z = -d_f/2$ plane. The corresponding vector potential inside the superconducting film is as follows:

$$\vec{\mathbf{A}}_{\rm M}(y) = -2Md_f \left[\arctan\left(\frac{2y+L}{d_f}\right) - \arctan\left(\frac{2y-L}{d_f}\right)\right]\hat{\mathbf{x}} \tag{1}$$

For a qualitative analysis consider first the case $L \ll \lambda_{\rm eff}$. This allows one to use London's equation neglecting the contribution to the vector potential from the screening current. Thus, the supercurrent is defined through the local vector potential generated by the ferromagnet:

$$\vec{\mathbf{j}}_s(y) = j_s(y)\hat{\mathbf{x}} = -\frac{c}{4\pi\lambda^2}\left[\vec{\mathbf{A}}_{\rm M}(y) + \vec{\mathbf{A}}_0\right] \tag{2}$$

where $\vec{\mathbf{A}}_0$ is a gauge term which can be found from the condition $\int_{-L/2}^{L/2} j_s(y)dy = 0$. Thus, this gives us simple analytical expression for spatial distribution of the screening current density:

$$\begin{aligned} j_s(y)/j_0 = M_0\Big[&\arctan\big((2y+L)/d_f\big) - \arctan\big((2y-L)/d_f\big) - \\ &2\arctan\big(2L/d_f\big) + (d_f/L)\ln\big(1 + 4L^2/d_f^2\big)\Big] \end{aligned} \tag{3}$$

Here we introduced a dimensionless magnetization $M_0 = 4\pi M d_f/\Phi_0$, where the value $4\pi M \approx 2\text{T}$ corresponds to $M_0 = 1$. Current density is expressed in terms of $j_0 = \Phi_0 c/8\pi^2 d_f \lambda^2$, where in SI units for $d_f = 30\text{nm}$ and $\lambda = \lambda_{\rm film}(0) \sim 220\text{nm}$ we have $j_0 \approx 17 \times 10^6\ \text{A/cm}^2$.

The opposite limit $L \gtrsim \lambda_{\rm eff}$ is of more interest in the system under consideration. The supercurrent screening the magnetic field in the S layer satisfies the London relation

$$\vec{\mathbf{j}}_s(y) = -\frac{c}{4\pi\lambda^2}\left[\vec{\mathbf{A}}_{\rm s}(y) + \vec{\mathbf{A}}_{\rm M}(y) + \vec{\mathbf{A}}_0\right], \tag{4}$$

$\vec{\mathbf{A}}_0 = -\int_{-L/2}^{L/2}\left(\vec{\mathbf{A}}_s + \vec{\mathbf{A}}_{\mathrm{M}}\right)dy/L$. is a gauge term that imposes $\int j_x(y)dy = 0$. The vector potential induced in the superconductor can be found from Biot-Savart's law:

$$\vec{\mathbf{A}}_s(\vec{\mathbf{r}}) = \frac{1}{c}\int \frac{d\vec{\mathbf{j}}_s}{R} = \frac{1}{c}\int \frac{\vec{\mathbf{j}}_s(\vec{\mathbf{r}'})d^3r'}{|\vec{\mathbf{r}} - \vec{\mathbf{r}'}|} \tag{5}$$

In the case of the thin superconducting film one has:

$$\vec{\mathbf{A}}_s(x,y) = \frac{d_s}{c}\int_{-L/2}^{L/2} dy' \int_{-l}^{l} dx' \frac{\vec{\mathbf{j}}_s(x',y')}{\sqrt{(x-x')^2 + (y-y')^2)}} \tag{6}$$

Consider a strip that is infinite in x direction with the length $l \to \infty$ in the presence of the supercurrent $\vec{\mathbf{j}}_s = j_s(y)\hat{\mathbf{x}}$. After integration the vector potential gains a simple form:

$$\vec{\mathbf{A}}_s(y) = -\frac{2d_s}{c}\hat{\mathbf{x}}\int_{-L/2}^{-L/2} \ln|y-y'| j_s(y')dy' \tag{7}$$

The last expression can be rewritten as an implicit equation for A_s:

$$\begin{aligned}\vec{\mathbf{A}}_s(y) = \frac{d_s}{2\pi\lambda^2}\hat{\mathbf{x}}\int_{-\frac{L}{2}}^{-\frac{L}{2}} \ln|y-y'|\Big(A_s(y') + A_{\mathrm{M}}(y')\Big)dy' - \\ \frac{d_s}{2\pi\lambda^2}\frac{1}{L}\hat{\mathbf{x}}\int_{-\frac{L}{2}}^{-\frac{L}{2}}\Big(A_s(y'') + A_{\mathrm{M}}(y'')\Big)dy'' \int_{-\frac{L}{2}}^{-\frac{L}{2}} \ln|y-y'|dy'\end{aligned} \tag{8}$$

This equation can be solved iteratively, starting with the ansatz $A_s(y) = 0$. The current distribution for both cases is shown in Supplementary Fig. S4. It is clearly seen that a simple analytical approach for $L \ll \lambda_{\mathrm{eff}}$ qualitatively gives a reasonable profile of the supercurrent.

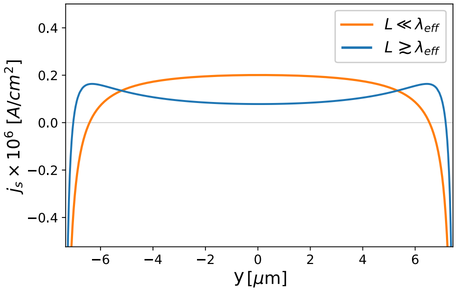

Supplementary Fig. S4 Screening current distribution across the S/F bilayer strip for parameters $L/d_f = 500$; $L/\lambda_{\mathrm{eff}} = 15$; $M_0 = 1$.

Transport Current Distribution

Here we present the analytic expression for the transport current distribution as obtained from a modification of the Bean critical state model [1, 2]:

$$j_x(y) = \begin{cases} \frac{2j_c}{\pi} \arctan\left(\sqrt{\frac{(L/2)^2-a^2}{a^2-y^2}}\right), & \text{if } |y| < a \\ j_c, & \text{if } a < |y| < L/2, \end{cases} \tag{9}$$

where we define the parameter $a = \frac{L}{2}\sqrt{1 - (\frac{I_t}{I_c})^2}$, which can be interpreted as half of the central field-free region. I_t is the total transport current and I_c and J_c are the critical current and the critical current density, respectively. In the calculation of the transport current distribution we have chosen the magnitude of the transport current to be close to what was used in the experiment ($I_t = 0.4$ mA and $I_c = 0.5$ mA), but it is evident that the model shows the same qualitative behavior for a wide range of current values.

We would like to elaborate now the sharp increase in the screening current density in the vicinity of the sample edge (Supplemetary Fig. S4). The reason for these peaks is due to the large value of the ratio $L/d_f = 500$. It is important to note that the model is not valid in the range $|y \pm L/2| < d_f$, which requires the introduction of a cutoff length scale for further investigation of j_s in this range. However, since the current value j_s at a distance $\sim d_f$ from the edge does not exceed the depairing current, $j_d \sim 200 \times 10^6 A/cm^2$ [3], this cutoff can be omitted for the purpose of our model.

It is also necessary to discuss the influence of vortices on the screening current density. The fact that the current j_s is large in the vicinity of the edges means that the stray field of the F layer may be able to induce vortices in this region. The zero-crossing of the current creates a potential well for a vortex, which results in the appearance of an equilibrium chain of vortices along the strip edge. Due to this confining potential, the large currents on the edge do not contradict the critical current j_c defined within the dynamic nature of the Bean model[1, 2]. Taking into account also the vortex supercurrent, j_v, would further reduce the sharp peaks along the edges, but due to the localized nature of the vortex chain, in the stationary regime the bulk value of the current (in the middle of the strip) should remain mostly unperturbed. For the aforementioned reasons, introducing a cutoff and/or adding j_v to the total current density would smooth the sharp peaks along the edge, but would not change the qualitative predictions of the model. In particular, the result that we get a different sign of j_{tot} in the bulk of the strip for opposite directions of j_t (which explains the diode effect) is robust to these additional considerations in the model.

Supplementary References

[1] Bean, C.P.: Magnetization of hard superconductors. Phys. Rev. Lett. **8**,

250–253 (1962). https://doi.org/10.1103/PhysRevLett.8.250

[2] Zeldov, E., Clem, J.R., McElfresh, M., Darwin, M.: Magnetization and transport currents in thin superconducting films. Phys. Rev. B **49**, 9802–9822 (1994). https://doi.org/10.1103/PhysRevB.49.9802

[3] Rusanov, A.Y., Hesselberth, M.B.S., Aarts, J.: Depairing currents in superconducting films of Nb and amorphous MoGe. Phys. Rev. B **70**, 024510 (2004). https://doi.org/10.1103/PhysRevB.70.024510
