# Supplementary material for: Direct observation of a superconducting vortex diode
Source: Nat Commun. 2023 Mar 24;14:1630. doi: 10.1038/s41467-023-37294-2 (PMC10036628; doi:10.1038/s41467-023-37294-2)
Supplement: Supplementary file 3 — Description of Additional Supplementary Files [file 41467_2023_37294_MOESM3_ESM.pdf]

Description of Additional Supplementary Files

File Name: **Supplementary Movie 1**

Description: **Transverse magnetic field H_y sweep correlating the vortex diode effect with the magnetic state of EuS**. **Left panel**: asymmetry factor $\Delta I_c = |I_c^+| - |I_c^-|$ as a function of transverse magnetic field H_y (blue symbols). The white diamond symbol represents the value of ΔI_c corresponding to the applied field in the images shown in the center and right panels. **Center panel**: SQUID-on-tip (SOT) images of the static out-of-plane component of the magnetic field $B_z^{dc}(x,y)$ emanating from the EuS/Nb bilayer under an applied transverse magnetic field H_y, as indicated in the left panel. **Right panel**: SOT image of the ac out-of-plane component of the magnetic field $B_z^{ac}(x,y)$ modulated with respect to an oscillating transport current $I_x^{ac} > I_c$ under an applied transverse magnetic field H_y. The value of the applied field is indicated in the left panel. The polarity of the signal depends on whether the magnetic feature appears in phase (blue) or at a π-phase (red) with respect to the oscillating current. Note that the images throughout the movie do not share the same color scale (see individual color bars). The magnetic field is swept down from above the saturation field $H_y > +H_s$, through the coercive field $H_y \sim H_c$ to below the negative saturation field $H_y < -H_s$. This is followed by a sweep up of the same range. These three field regions cover the different states of the diode effect (i.e., $\Delta I_c > 0$, $\Delta I_c \sim 0$, $\Delta I_c < 0$) as portrayed in the left panel. The field values cover a range of -16 mT $< H_y <$ 16 mT.

File Name: **Supplementary Movie 2**

Description: **Longitudinal magnetic field H_x sweep correlating the vortex diode effect with the magnetic state of EuS**. **Left panel**: asymmetry factor $\Delta I_c = |I_c^+| - |I_c^-|$ as a function of longitudinal magnetic field H_x (red symbols). The white diamond symbol represents the value of ΔI_c corresponding to the applied field in the images shown in the center and right panels. **Center panel**: SQUID-on-tip (SOT) images of the static out-of-plane component of the magnetic field $B_z^{dc}(x,y)$ emanating from the EuS/Nb bilayer under an applied longitudinal magnetic field H_x, as indicated in the left panel. **Right panel**: SOT image of the ac out-of-plane component of the magnetic field $B_z^{ac}(x,y)$ modulated with respect to an oscillating transport current $I_x^{ac} > I_c$ under an applied longitudinal magnetic field H_x. The value of the applied field is indicated in the left panel. The polarity of the signal depends on whether the magnetic feature appears in phase (blue) or at a π-phase (red) with respect to the oscillating current. Note that the images throughout the movie do not share the same color scale (see individual color bars). The magnetic field is swept down from above the saturation field $H_x > +H_s$, through the coercive field $H_x \sim H_c$ to below the negative saturation field $H_x < -H_s$. This is followed by a sweep up of the same range. It is evident that the diode effect vanishes (i.e., $\Delta I_c \sim 0$) for all longitudinal field regions, as portrayed in the left panel. The field values cover a range of -16 mT $< H_x <$ 16 mT.
